# The Cost of Love: Emotional Labour and Moral Tensions in the Lives of Chinese Young Carers

**DOI:** 10.1111/1468-4446.70070

**Published:** 2025-11-29

**Authors:** Kefan Xue, Kaidong Guo

**Affiliations:** ^1^ Oxford School of Global and Area Studies University of Oxford Oxford UK; ^2^ Social Research Institute UCL London UK

**Keywords:** China, emotional labour, filial piety, informal caregiving, young carers

## Abstract

Like adults, children also provide care. This article explores the emotional labour of young carers who care for ill or disabled family members in China, a context where children's caregiving remains largely invisible in both policy and scholarship. Drawing on 15 months of ethnographic fieldwork with 30 young carer families in both urban and rural China, this study investigates how children manage, suppress, and perform emotions to sustain family life in the absence of formal welfare support. Building on Hochschild's (2012) concept of emotional labour, the analysis reveals how culturally embedded scripts, such as filial piety, operate as ‘feeling rules’ that legitimise and normalise children's affective contributions. Findings demonstrate the ambivalent nature of children's informal caregiving: while caregiving can foster pride, maturity, and recognition, it also generates exhaustion, guilt, and role confusion, extending beyond conventional notions of parentification. Situating these dynamics within the political economy of care, the study shows how children's emotional labour operates as an unacknowledged subsidy to social reproduction, masking structural inequalities and welfare retrenchment. By recognising children as consequential emotional actors, the article reconceptualises care as both a moral practice and a site of inequality, advancing young carers scholarship beyond Global North contexts.

## 1 Introduction

In recent decades, a growing body of international research has documented the experiences of children under 18 years old who undertake caregiving responsibilities for ill or disabled family members, commonly referred to as ‘young carers’ (Becker 2000). Regarding the prevalence of young carers, no official statistics are available in China, and the group remains absent from both government reporting and large‐scale academic surveys. In contrast, studies from advanced industrialised societies estimate that between 2% and 8% of children and young people undertake regular caregiving responsibilities, with variation depending on methods of measurement (Leu et al. 2019). Despite their presence across diverse contexts, including European countries such as the UK, Norway and Sweden, as well as the United States, Canada, Australia, and Japan (Joseph et al. 2020). Yet in China, children's unpaid caregiving remains largely invisible in policy, research, and public discourse. This invisibility stems not only from the private nature of their caregiving, typically framed as acts of love rather than child labour (Akkan 2020), but also from dominant imaginaries of childhood as a time of innocence, dependence and emotional purity (Becker 2007). These imaginaries sustain a binary between the cared‐for child and the caregiving adult, leaving little conceptual space for children's labour and moral responsibility (Gowen et al. 2022; Ruiz 2024). Consequently, young carers occupy a paradoxical position: while their everyday practices are essential to family survival, their labour and voices remain marginal in scholarship and institutional recognition (Rose and Cohen 2010; Schweiger 2025).

Although scholarly attention to young carers has grown, much of the literature still focuses on categorising caregiving tasks and assessing measurable impacts, such as educational disruption, emotional stress, or social isolation (Clay et al. 2016; Stamatopoulos 2018). Emotions such as guilt, pride, and resentment are acknowledged but often treated as incidental, with little examination of their relational and sociocultural dimensions (Skovdal et al. 2009). This tendency flattens the emotional complexity of caregiving, sidelining affect as a core component of the moral and relational dynamics of care. Even when framed through concepts such as parentification or role reversal (Earley and Cushway 2002), these experiences are predominantly cast in developmental or pathological terms (Rose and Cohen 2010; Stamatopoulos 2018), overlooking how emotional labour is shaped by familial power relations, cultural expectations, and normative feeling rules.

This article addresses that gap by offering a sociologically informed account of young carers' emotional labour, viewing it not merely as a private burden but as a moral practice shaped by structural constraints and culturally embedded feeling rules. In China, research on children's caregiving remains scarce. However, studies on intergenerational family relations have shown that care practices are profoundly shaped by deeply rooted cultural norms, including *xiao* (filial piety), *dongshi* (being sensible and thoughtful), *chiku* (enduring hardship),^1^ and *baoen* (reciprocal gratitude), which function as powerful ‘feeling rules’ governing intergenerational life (Gu 2022a; Murphy 2020). Children who care for elders or ill relatives are praised as *dongshi*, and their actions are framed as moral virtues rather than as responses to concrete needs (Gruijters 2017). Such expectations normalise and moralise emotional labour, muting expressions of hardship and valorising responsibility. In families where adult support is unstable, these ideals can obscure the affective costs of care (Rai 2024; Schweiger 2025), and help justify the lack of state welfare, shifting care responsibilities from public provision to families, and crucially, onto children (Schweiger 2025), thereby reinforcing structural inequities (Wollner 2019).

Following Hochschild (2012), emotional labour refers to managing and expressing feelings in ways that align with socially prescribed norms, a concept later extended by feminist scholarship to intimate and care contexts. Yet few studies have applied this lens to examine how young carers are shaped by, and contribute to, culturally specific ideals of love, duty, and endurance. In the Chinese context, children's emotional work is often romanticised as innate virtue or familial attachment, rather than recognised as a situated practice shaped by cultural, gendered, and policy environments (Schweiger 2025). This study builds on Hochschild's insights to argue that young carers operate within an emotional framework that demands not only caregiving but the cheerful performance of sacrifice, making emotional labour both a mode of care and a process of moral subject formation.

In doing so, this study draws on emotional labour (Hochschild 2012) to examine how children in China navigate caregiving under moral obligation, and emotional restraint. Specifically, the research question is: How are the experiences, performances, and negotiations of emotional labour co‐constructed within caregiving relationships involving children under 18 (i.e., young carers) in China, and how do the views of care recipients and other family members contribute to understanding these morally charged dynamics? Emotions are treated not as private psychological states but as relational practices embedded in culturally specific feeling rules, sustaining intergenerational solidarity in the absence of institutional support. This perspective moves beyond notions such as role reversal and parentification (Earley and Cushway 2002), situating children's caregiving within wider social and political contexts, and recognising emotional labour as both adaptive coping and a process of moral subject formation.

By positioning emotional labour as both culturally meaningful and structurally consequential, this study makes three contributions. First, it extends the concept of emotional labour into the underexplored terrain of intergenerational caregiving by children, showing how affective work is shaped by moral obligation, power asymmetries and culturally embedded feeling rules, and contributing to sociological understandings of care, moral subjectivity and children's agency. Second, drawing on 15 months of ethnographic fieldwork with 30 young carer families in China, it provides an empirically rich account of how emotional labour is both expected and legitimised through cultural idioms of filial duty and emotional endurance. Third, it demonstrates how these affective and moral discourses act as normalising mechanisms, masking structural inequalities and shifting care responsibilities from public provision to families and, crucially, to children, thereby depoliticising and privatising care. The article proceeds with a review of relevant literature, an outline of methodology, presentation of findings on three patterns of emotional practice, and a discussion of theoretical implications for understanding moralised care regimes. In doing so, the article also contributes to ongoing debates within the political economy of care, which frame care as both a moral practice and a site of inequality (Petterson 2012; Tronto 2020).

## 2 Literature Review

### 2.1 Approaching Young Carers in Contemporary China

Although existing literature in China does not explicitly investigate young carers, the figure of children who care and contribute to the family by undertaking a range of domestic responsibilities has appeared in monographs (Murphy 2020), journal articles (Gruijters, 2017; Ye and Pan 2011), and a non‐fiction novel (Huang 2020). For example, Murphy (2020) highlights how left‐behind children^2^ in China frequently take on caregiving roles for ill or elderly family members, often balancing domestic tasks with agricultural duties. Similarly, Gruijters (2017) notes that in multi‐generational rural households, grandchildren often step into primary caregiving roles when neither a spouse nor an adult child is available, fulfiling these duties under the moral framework of filial piety.

As mentioned in the Introduction, filial piety plays a foundational role in the Chinese moral system, where children are expected to demonstrate unwavering respect, obedience, and care for their elders (Murphy 2020). Despite the core role of filial piety in contemporary Chinese society, its practice has evolved in response to significant socio‐economic and political transformations. Since the onset of China's market‐driven economic reforms in the late 20th century, the expectations surrounding filial obligations have shifted. Power within the family, traditionally held by older generations, has gradually shifted to younger, more educated, and economically advantaged generations (Yan 2016).

This shift has led to a more child‐centred family model, emphasising emotional attachment and academic success over the traditional focus on economic security (Goh and Kuczynski 2014). However, the transformation from economic to emotional investment does not imply the diminution of filial obligations. Parents still expect their children to reciprocate care, not necessarily through material support, but by excelling in education and fulfiling familial moral expectations (Gu 2022a). This expectation is especially pronounced in the context of migration, where migrant parents endure hardship (*chiku*) for the sake of their children's educational success, framing their sacrifices as investments in the children's future (Gu 2022a; Murphy 2020). Despite these socio‐cultural shifts, filial piety remains a vital aspect of family dynamics, particularly in rural regions, where (adult) children continue to bear significant caregiving responsibilities (Murphy 2020). This emphasises the resilience and transformation of cultural values within contemporary Chinese families.

Thus, while filial piety and *chiku* remain cultural cornerstones, they generate a distinctive set of emotional expectations and challenges for children. For example, for young carers, these values may intersect to shape their caregiving experiences, requiring them to navigate complex emotional labour that is frequently overlooked or undervalued. Understanding these cultural norms is essential to interpreting the emotional labour young carers experience in China, as they balance familial roles, filial piety, caregiving responsibilities, emotions, and moral imperatives.

### 2.2 Theoretical Framework: Emotional Labour

Emotional labour provides a powerful lens for interrogating the moral, cultural and structural dimensions of young carers' experiences in China. Originally theorised by Hochschild (2012) in the study of commodified service work, the concept has since been extended to a wide range of care and interpersonal contexts, including nursing, teaching, counselling and childcare (Boyer et al. 2013; Ruiz 2024). At its core, emotional labour refers to the regulation of one's own emotions and the orchestration of others' emotions in accordance with socially embedded ‘feeling rules’ that prescribe what is appropriate to feel and display in a given context. More recently, research has highlighted its salience within families where emotional labour underpins the reproduction of relationships and moral orders (English and Brown 2023).

Despite this conceptual broadening, research applying emotional labour to caregiving has overwhelmingly centred on adult perspectives, particularly mothers, paid care workers and other adult family members (English and Brown 2023; Fairchild and Mikuska 2021). Children's emotional contributions, when acknowledged at all, are often subsumed under notions of ‘help’ or ‘love’ rather than analysed as labour (Ruiz 2024). In the Chinese context, this gap is especially striking. Existing studies show that children frequently support family members through domestic work or academic achievement (Gu 2022a; Murphy 2020), but they seldom address children's management of feelings, such as concealing distress, performing gratitude or sustaining family harmony, as analytically significant forms of labour. These emotional practices are deeply shaped by cultural norms such as *xiao* (filial piety), *dongshi* (being sensible and thoughtful), *chiku* (enduring hardship) and *baoen* (reciprocal gratitude). Such values create a moral economy in which children's willingness to absorb emotional strain is legitimised as love and duty (Gu 2022a; Murphy 2020), even as this labour obscures the structural absence of welfare provision (Schweiger 2025). Recognising children as emotional labour is therefore not only an analytical reframing but also a step towards unsettling entrenched binaries between caring adults and cared for children.

This study positions emotional labour as a central analytical framework because it captures the intersection of cultural norms, moral obligations, and structural inequalities that shape young carers' experiences in China. By conceptualising children's caregiving not merely as spontaneous affection but as labour governed by culturally specific feeling rules, this approach foregrounds the relational agency (Spyrou et al. 2019) through which children negotiate care asymmetries. It also highlights how affect operates ideologically, legitimising the redistribution of care responsibilities from the state to families, and within families, onto children (Schweiger 2025). In doing so, the framework makes visible children's emotional practices as consequential forms of labour that both sustain and, at times, contest the moral economy of care.

## 3 Methodology

This article draws on research undertaken as part of Author 1's doctoral study at the University of Oxford. The study involved 15 months of ethnographic fieldwork conducted between 2022 and 2024, exploring the lived experiences of young carers in China through in‐depth qualitative interviews and participant observation. Fieldwork was carried out in two contrasting sites within Zhejiang Province: the urban district of L and the rural county of C, allowing for comparison across different socioeconomic and cultural caregiving environments.

### 3.1 Sampling and Recruitment

Purposive maximum‐variation sampling (Daniel 2012) was adopted to capture diverse caregiving contexts. Families were eligible if at least one child regularly provided care for a relative with a chronic illness, disability, or mental health condition. Variation was sought across gender, family composition, duration of care, and type of illness. Participants were recruited through local sub‐district offices, village committees, social workers, and schools that identified suitable families. During preliminary visits, the researcher accompanied these professionals to introduce the study and establish rapport. Of the 42 families approached, 30 agreed to participate. Recruitment ceased when thematic saturation was reached, meaning that no new insights emerged from additional cases (Small 2009). This process ensured representation of both urban and rural caregiving situations while balancing feasibility with depth.

### 3.2 Participants

The study involved 60 participants: 30 young carers and one family member from each household, usually a parent, grandparent, or sibling. The young carers were aged between 12 and 17 and had been providing care for two to 12 years. The sample was gender‐balanced, comprising 15 girls and 15 boys. All participants were attending school during the study period: 13 were in junior secondary and 17 in senior secondary education, including 15 in vocational schools. Families varied in structure, with 16 being single‐child, fourteen multi‐child, and fifteen lone‐parent households. Some young carers shared caregiving responsibilities with extended family members, offering insights into how caregiving dynamics shifted according to household composition.

Care recipients experienced a wide range of conditions, including diabetes, renal failure, disabilities, speech impairments, and mental illnesses, often co‐occurring. Seventeen families were classified as low‐income, six as marginally low‐income, and seven as financially constrained, with poverty particularly visible in rural areas reliant on farming or casual labour. Only eight families received support from extended kin, and one employed a part‐time carer. Together, these cases illustrate the economic precarity and familial interdependence shaping children's caregiving. Further demographic and contextual details of participants are provided in Appendix I.

## 4 Data Collection

### 4.1 Semi‐Structured Interviews

Sixty semi‐structured interviews were conducted: one with each young carer and one with their corresponding family member. Interviews were held primarily in family homes, although in several cases participants requested community centres or school offices to ensure privacy. Each interview lasted between 60 and one hundred and 20 minutes and was audio‐recorded with consent. Interviews with young carers explored the emotional, social, and moral dimensions of caregiving—what they did, how they felt, and how they interpreted their roles. Interviews with family members examined perceptions of children's contributions and relational dynamics within the household. Conducting interviews separately allowed participants to speak freely about sensitive topics and helped to mitigate power imbalances between children and adults.

### 4.2 Participant Observation

Participant observation complemented the interviews by capturing embodied caregiving practices and everyday affective exchanges. In L District, two weekday (7 a.m.–9 p.m.) and one weekend visit per family were conducted, while in C County visits took place mainly at weekends because many children boarded at school. During the 2023 summer break, when children were at home full time, rotational visits across both sites enabled longitudinal observation of the seasonal rhythms of care.

Observations focussed on daily routines, household chores, emotional support, and schoolwork. Direct personal care (e.g., bathing or toileting) was observed only when families explicitly invited my presence; in such cases, additional care was taken to ensure privacy and comfort. I also participated in household tasks such as cooking, cleaning, and assisting with errands. While this participation facilitated rapport and offered an insider's perspective, I remained reflexive about how my involvement might influence family dynamics. Fieldnotes and reflexive diaries documented these interactions and my efforts to balance participation with observation. Although visits were lengthy, families generally described them as supportive rather than intrusive, appreciating the opportunity to share their experiences in depth. Where concerns about intrusion arose, arrangements were discussed openly and adjusted accordingly.

### 4.3 Data Analysis

The dataset comprised 60 interview transcripts, fieldnotes, and reflexive diaries. All interviews were transcribed verbatim in Mandarin, with local dialects manually corrected for accuracy. Analysis followed a thematic approach that combined inductive and deductive reasoning (Boyatzis 1998).

First, transcripts were read repeatedly to identify recurrent emotions, practices, and moral discourses. Open codes were then generated to capture patterns such as ‘emotional suppression’, ‘reluctance and guilt’, and ‘sources of pride’. Second, through constant comparison within and across cases, these codes were condensed into broader categories. Third, the categories were synthesised into three overarching themes: Emotion under Strain, Trapped in Duty, and Ambivalent Rewards, which structure the Findings section. NVivo 12 was used for data management, while reflexive memos helped to interrogate how interpretation evolved. Triangulation across interviews, observations, and diaries enhanced analytical credibility, and cross‐checking between children's and adults' narratives illuminated relational tensions. Quotations were selected to illustrate both typical and divergent cases, ensuring that the findings were grounded yet nuanced.

### 4.4 Ethics and Reflexivity

Informed consent was obtained from all participants, with parental permission secured for minors. Owing to local sensitivities, oral consent was preferred to written forms; statements were audio‐recorded and stored securely. Ethical approval was granted by the University of Oxford Central University Research Ethics Committee (Ref. R82187/RE001). Although China lacked formal local ethics review structures, permission to conduct the research was obtained from district and village authorities as well as participating schools.

Ethical vigilance was maintained throughout the research process. Families were reminded that participation was voluntary, and interviews or observations were paused whenever distress occurred. All names used are pseudonyms. Sensitive events, such as emotional outbursts, were handled with care, and privacy was ensured. Besides, as a young Han Chinese woman fluent in Mandarin, the researcher shared cultural and linguistic familiarity that facilitated trust. However, her position as a doctoral student from an elite UK institution also introduced elements of class and institutional distance. Reflexive diaries were used to examine how these positionalities shaped recruitment, interpretation, and family interactions, recognising that ethnographic knowledge is co‐produced rather than extracted.

## 5 Findings—Young Carers as the Emotional Labour of Love

The findings from this study show that children undertook a complex mix of caregiving tasks that Chinese adults do not usually expect of children, as suggested by existing scholarship (Goh and Kuczynski 2014). Compared to ‘non‐caregiving’ children in China, who may also be involved in doing domestic chores and sibling care to a limited extent (Goh and Kuczynski 2014), children in this study undertook a substantial range of caregiving and household responsibilities,^3^ personal and intimate care,^4^ among others, as the substitute labour force for adult family members, which led them to engage in the process of emotional labour (Hochschild 2012) and parentification (Earley and Cushway 2002).

By listening to young carers' and their families' voices and observing the dynamics of their family lives, in this section, we focus specifically on the emotional landscape of young carers and the emotional labour that they experience. In what follows, we explore young carers' emotions at home and how they manage conflicted emotions to fulfil their caregiving responsibilities, filial obligations, and maintain their family's harmony. Overall, findings show that young carers engage in emotional labour, including constantly enduring, managing, and hiding feelings such as *weiqu*,^5^ frustration, guilt, and affection in order to meet social and moral expectations. Their emotional work involves not only protecting others but also navigating their own identities as children and caregivers.

### 5.1 Emotion Under Strain: Children Negotiating Roles and Responsibilities

In this study, young carers' responsibilities often extended beyond physical tasks to include emotional care, such as monitoring the care recipient's emotional and mental state, accompanying and comforting them, and trying to cheer them up—work that frequently involved significant emotional labour. This was particularly true for children who, while fulfiling caregiving roles, had to manage their own emotions to meet familial and societal expectations (Hochschild 2012). For example, Qingyu, a 15‐year‐old secondary carer providing care for her father with chronic glomerulonephritis for six years, described intentionally suppressing her emotions to manage caregiving efficiently:

I just tried to turn my emotions off so I can do all these [caregiving] in a more organised way and won’t raise other people’s concerns… I guess I have developed a new character setting when facing my parents. Because I don’t really want them to worry about me, and I don’t want to waste time explaining [about my emotions].

Qingyu's experience illustrates how young carers balance their own emotional responses with the needs of others, within familial and cultural expectations (Hochschild 2012). Her mother, meanwhile, quietly noted during interview that ‘She never lets us see her upset [pause], which makes me feel both easy and uneasy.’ Read together, these perspectives highlight how children's suppression of feelings was not simply self‐discipline but part of a tacit family arrangement in which parents drew reassurance from children's composure while also sensing its costs.

Many young carers similarly regulate emotions according to culturally embedded ‘feeling rules’ (Hochschild 2012), often hesitating to openly discuss a family member's illness for fear of causing distress. Despite this, they proactively seek information online and learn from others' caregiving experiences, reflecting a desire to provide informed, compassionate care while managing their own emotions and supporting the emotional well‐being of the family. For example, Chenxuan, a 15‐year‐old girl from L District caring for her grandfather with terminal uremia after her father disappeared, explained:

At home, we rarely talk straight about illness or diagnosis‐related things, so when I cared for my grandfather I need to take care of this “avoidance” emotion at the same time [laughs]… I usually just, just provide care quietly without saying much.

Her grandfather (the care recipient) similarly remarked: ‘We don't say too much to the child. Why scare her? It's already too much.’ This mutual avoidance underscores how silence itself became a form of shared emotional labour: children and adults jointly maintained a fragile equilibrium by keeping distress unspoken, even as it weighed heavily on them.

Yet emotional outbursts occur when emotional labour becomes overwhelming. Yong, a polite and quiet 17‐year‐old primary carer in C County, was about to finish high school. His father was diagnosed with AIDS over a decade ago, followed by his mother. In recent years, both parents' health deteriorated—his mother, Ms Mei, developed cervical cancer in 2017 and thyroid cancer in 2018—leading to increased caregiving and financial burdens. Ms Mei frequently expressed feelings of *weiqu*, believing her HIV infection resulted from her husband's disloyalty, often directing her resentment towards Yong. During visits, Yong rarely shared personal feelings, quietly listening and occasionally soothing her. Sometimes his coaxing worked; sometimes it did not. On one occasion, I witnessed him shout: ‘Your marriage is your choice, mum! If you can't bear all this, why don't you just divorce him? I can't continue playing the role of your husband anymore!’

Yong's frustration illustrates the emotional toll of caregiving, extending beyond the typical concept of parentification (Earley and Cushway 2002). As Chase (1999), p. x) notes, ‘Parentified children, in fact, are parents to their parents and fulfil this role at the expense of their own developmentally appropriate needs and pursuits.’ Yong suggested he was playing the role of ‘a husband’ to his ill mother, highlighting how adult‐child caregiving can involve other forms of role transposition (Aldridge and Becker 2003). This exhaustion and role confusion demonstrate the complexity of caregiving when responsibilities stretch beyond typical parent‐child boundaries, where emotional labour is both morally compelling and personally depleting.

Significantly, Ms Mei herself reflected on this inversion of roles: ‘I feel quite awkward; I place some husband‐like requirements on him if I must say it clearly. But I don't know what to do. [silence] There is no way out [for me to express feelings].’ Her recognition did not erase Yong's burden, but it did reveal how both mother and son struggled with the intimacy and constraint of their emotional bond. In a private conversation, he shared:

I know I shouldn’t talk to her like that, and she must feel very hurt. … Her marriage is her choice, but you know … I feel like she [mum] has been morally abducting me, and she wants too much emotional value… I’m exhausted … Can I walk away and disappear? [pause] the answer is I can’t.

In this context, Yong's exhaustion, frustration, and sense of *weiqu* clashed with guilt and love for his mother. These contradictions demonstrate that emotional strain was not borne by Yong alone but negotiated between him and his mother, in a cycle where her dependence and guilt intensified his sense of entrapment. Despite feeling unseen and exhausted, Yong continues to provide care because the relationship is both morally compelling and practically unavoidable.

Like Yong did, young carers' devotion to caregiving practices and emotional labour has overwhelmed them. Similar emotional outbursts were observed in many young carer families, including Yifan's. Yifan, a 16‐year‐old girl from C County, has been providing care for her father, who suffers from thrombopenia, hypohepatia, and chronic renal insufficiency. Once, she accused her father: ‘Do you think that I must do all these [caregiving]? Why does everyone take these all for granted?’ Following these words, Yifan's father fell silent and left the room. Later, however, he admitted feeling both shame and helplessness, remarking that ‘No father wants to see his child like this, but our family, really [pause], we have no choice.’ This moment raises important questions about how care recipients and other adult family members perceive young carers' emotions. Findings from this study indicate that while family members may not always fully articulate their understanding, there is a certain level of understanding and empathy towards the emotional struggles of young carers. When children express their distress or frustration, care recipients and adult family members often experience a sense of guilt or sorrow.

For example, Chao, a 16‐year‐old boy in a lone‐parent household, has been providing care for his mother, who was first diagnosed with breast cancer in 2019. Although she underwent surgery, the cancer recurred in 2023. Ms Wen, Chao's mother, reflected on her son's emotional turmoil: ‘I know he [Chao] was terrified and lived in fear every day when I first got sick [the breast cancer], even after it was removed.’ In this case, the mutual emotional exchange between Chao and his mother is part of the complex emotional labour that characterises young carers' experiences. Such mutual emotional surveillance and care reflect the deep emotional ties that underpin caregiving, which is not merely an act of duty but a way of negotiating and coping with the anxiety, fear, and love inherent in the caregiving relationship.

### 5.2 Trapped in Duty: Guilt, Reluctance, and Jinli^6^

As the question raised by Yong above suggests, can young carers walk away and disappear? Also, do young carers themselves want to walk away and disappear? In this study, most young carers presented inner struggles and conflicting emotions regarding their caregiving roles and relationships with the care recipients. As mentioned before, engaging in caregiving practices, including emotional labour, became one of the most accessible ways for children as minors to demonstrate their love and filial piety to the care recipients and other adult family members, echoing existing studies (Gu 2022a; Stamatopoulos 2018). Young carers manage their emotions by performing emotional labour, which involves regulating and suppressing their own feelings to meet the expectations placed on them. As the 12‐year‐old young carer Xiyao, who lived in a lone‐parent household and had been caring for her mother with a congenital speech disability for eight years, said:

If I complain to anyone about this [caregiving], I’m afraid that people would misunderstand me as someone who “dislikes” my disabled mum … I think it’s natural for me to care for her if she can’t speak, right? If not me, who else can do this?

Her grandmother, however, offered a different angle: ‘Sometimes I worry she feels stuck, but she never complains in front of her mother. I tell her that even children need rest.’ Read together, these voices reveal how a child's sense of inescapable duty intersected with an elder's recognition of her burden. This interplay shows how reluctance, guilt, and responsibility were not carried by children alone but embedded in wider family negotiations.

Reluctance often gave way to guilt after moments of conflict. Only a day after Yong argued with his mum, he told me that he regretted raising the confrontation. In his words, losing his temper was incredibly immature and should not have happened. Yong fell into deep self‐blame and said: ‘I regret that I complained so much about my parents. I feel like I'm terrible [pause] I don't know how to put it … My mum is really, really unwell sometimes, but she does her best to raise me.’

Young carers' inner struggles were intensified when they doubted the adequacy of their caregiving. Because care recipients were loved ones, children sought to give their best. The Chinese term *jinli*, meaning to devote one's utmost effort, emerged as a key theme in exploring children's feelings about caregiving. e.g., Xingxing, a 17‐year‐old primary caregiver from L District, had been caring for her foster mother, who suffered from epilepsy and a mental disorder, since age five. She lost her foster father to cancer at eight. Despite her efforts to balance caregiving with study, interests, and other aspects of life, she sometimes questioned whether she had been sufficiently *jinli*:

I think I have [*jinli*], but, I mean, sometimes, I would be lazy. Sometimes, I wanted to escape [from caregiving]. … When mum sometimes screamed at night, I pretended I didn’t hear … Well, I don’t think I have the qualifications to complain too much.

Xingxing's pretending not to hear reflects more than avoidance; it reveals the tension between her emotional limits and her desire to be a ‘good’ daughter and ‘responsible’ caregiver. Her guilt for not always being attentive shows how caregiving is emotionally contradictory and consuming. Indeed, there is no clear benchmark for determining whether a child has demonstrated *jinli* in caregiving, particularly in cases of incurable illness, which makes children's feelings about caregiving even more complex.

In rural C County, caregiving also extended into farm work essential for family survival, sometimes involving tasks inappropriate and dangerous for children. Xiang, a 15‐year‐old boy, balanced his excitement about entering senior secondary school with worries about his grandparents' dependence on him for both farming and care. His grandmother, explained with tears:

His grandfather couldn’t work any [sigh]. So if he couldn’t help me with farming, I’m too old to do so much [pause], and our whole family will starve to death [silence] … We have no money, and I’m worried I won’t be able to afford his college tuition. Could you help us? [pause] Please help us …

Xiang echoed these anxieties, said:

My grandfather’s body is heavy … grandmother won’t be able to support him every day and may even hurt her knees [sigh] … I’ll go back home immediately after every Friday’s schooling and do farming as much as I can during the weekend.

This account shows how caregiving responsibilities were inseparable from household survival. While Xiang felt pride and anticipation about his schooling, he also carried guilt about leaving his grandparents behind. For his grandmother, pride in Xiang's academic achievement was entwined with guilt and anxiety about depending on him. Here, duty was mutually felt across generations, highlighting how the pursuit of *jinli* was relationally negotiated within a context of scarcity and survival.

Overall, struggles with guilt, reluctance, and the pursuit of *jinli* demonstrate that caregiving was not simply a practical duty but a relational negotiation. Emotional labour involved cycles of suppression, self‐blame, and reassurance, in which children feared falling short and adults oscillated between gratitude and guilt. These dynamics reveal caregiving as both morally compelling and emotionally consuming, embedded in the layered ties of family life.

### 5.3 Ambivalent Rewards: Pride, Recognition, and Constraint

Although earlier sections have highlighted the strains and contradictions of caregiving, young carers' emotional experiences were not solely negative. Caregiving also generated moments of pride, growth, and recognition. Many young carers reported personal development through their responsibilities. As Rai (2024), 141) notes, some children ‘gain from being a carer,’ reflecting the experiences of participants in this study. Several described feeling more mature, contributing meaningfully to their families, and developing closer bonds with family members. They also expressed pride in their caregiving and a sense of being irreplaceable, echoing previous research highlighting caregiving as a source of pride, responsibility, and connection (Clay et al. 2016).

e.g., Liang, a 16‐year‐old from L District caring for his grandmother and younger brother after the deaths of his father and grandfather, described taking on nearly all physically demanding or repair‐related tasks in the household. He expressed pride in fulfiling adult responsibilities:

I didn’t realise that when dad was around [silence] so many things needed to be checked [pause] and maintained regularly. Now I do it myself [silence] and there are moments when I feel like I am the man, the head of the family …

Young carers often gain recognition from their families and the wider community (e.g., neighbours, social workers, teachers) as being thoughtful (*dongshi*), filial, mature, or robust. Liang's mother proudly described him as the only ‘man of the house,’ filial since his father and grandfather's illness. During my visits, neighbours praised Liang, suggesting he practised the traditional virtue, ‘the eldest brother is like a father’ (Liu 2023). Yet, such recognition itself demanded emotional labour, as Liang was expected to outwardly accept pride and gratitude while internally questioning his ability to sustain these responsibilities. In private conversations, Liang revealed his inner struggle. While proud of being the ‘man of the house,’ he felt constrained by caregiving responsibilities and increasingly lost control over his own life and aspirations, wishing to escape. He said tearfully: ‘I don't want to be filial anymore … *Jiejie* [sister, referring to me], can I just be selfish? But doing this [be selfish and don't be filial] is also too difficult.’

Other young carers expressed similar reluctance when praised as *dongshi* or for enduring hardships (*chiku*). For example, Mengyuan, a 17‐year‐old left‐behind^7^ child living with grandparents, caring for a grandmother with multiple chronic illnesses and a grandfather with dementia and physical disability, said: ‘When people say I'm *dongshi*, it seems like I have been constrained in the framework of being a “good girl”. They just hanged me there [in that framework].’ Her account illustrates how moral labels such as *dongshi* or ‘good girl’ simultaneously confer recognition and impose constraints, fixing children within normative scripts that limit the legitimacy of ambivalence or refusal.

In summary, caregiving exposes young carers to an emotionally ambivalent journey: pride and maturity coexist with exhaustion and self‐doubt. Recognition and praise themselves became sites of emotional labour, as children learned to accept or perform them outwardly while negotiating ambivalence privately. This ambivalence is not accidental, but produced at the intersection of cultural expectations of filial piety and the structural absence of welfare support. Together, these forces generate a moral economy in which children's emotional labour is both legitimised and demanded, shaping caregiving as a deeply contradictory experience.

## 6 Discussion

This study reveals a paradox at the heart of young carers' everyday lives: their emotional labour is celebrated as a sign of virtue, often framed through *dongshi* (being sensible and thoughtful) and filial piety, yet it arises precisely from and helps sustain the structural absence of public welfare. In China, where care for dependent family members is culturally legitimised but minimally supported by formal institutions (Gu 2022b), children undertake caregiving responsibilities that are moralised as acts of love and duty. As one participant, Xiyao, remarked, “If not me, who else can do this?”, a sentiment that affirms family solidarity while obscuring the structural inequalities that necessitate such labour (Schweiger 2025; Wollner 2019). This paradox invites a rethinking of emotional labour in contexts where the moralisation of care masks the state's withdrawal from welfare provision.

### 6.1 Moral Internalisation

The findings indicate that young carers in China absorb culturally specific feeling rules (Hochschild 2012) that require them to suppress frustration, hide resentment, and display gratitude in order to preserve familial harmony. These rules are grounded in social norms of *chiku* (enduring hardship) and self‐sacrifice, and are often expressed through *weiqu*, a culturally resonant emotion blending grievance, sadness, and moral restraint (Zou et al. 2020). The case of Yong, who listened in silence to his mother's long accounts of marital betrayal before finally bursting out that he ‘could not play the role of her husband anymore’, illustrates how *weiqu* is both a burden and a badge of worth. By enduring such feelings without complaint, young carers affirm their moral standing within the family, even when the demands far exceed what is developmentally appropriate. This pattern echoes Pettersen's (2012) notion of altruistic care, where the ethical and political dimensions of caregiving are obscured by its framing as selfless devotion. Through such internalisation, emotional labour becomes embedded in a moral economy in which the costs of care remain within households, borne most heavily by children rather than being redistributed through collective provision. As Oydgard (2017) observes in the context of informal caregiving, normative discourses of duty create a sense of inevitability, compelling individuals to step in where formal systems fail.

Notably, although this article has focussed primarily on children's emotional labour, the findings also reveal the relational and reciprocal nature of emotion work within families. Parents were not passive recipients of care but also engaged in emotional labour to protect and reassure their children, often concealing their own distress or guilt in order to maintain stability. As seen in mother Mei's and Wen's reflections, adults actively managed their emotions to reduce the emotional burden on their children, even when constrained by illness and poverty. These mutual affective negotiations suggest that emotional labour circulates within families rather than residing in one actor alone, highlighting the need for future research to examine how intergenerational emotion work both reproduces and, at times, mitigates structural inequalities in care from the perspectives of different family members.

In China, this sense of inevitability is amplified by public discourses that romanticise children who provide care as *dongshi* (being sensible and thoughtful) and selfless, turning acts such as Liang's repair of household appliances for his grandmother into moral exemplars of filial responsibility. This romanticisation is vividly reflected in Chinese mainstream media. Reports such as ‘An Eight‐Year‐Old Girl Cares for Her Paralysed Mother for 14 Years without Leaving Her Side’ (People’s Dail and y Online 2014) and ‘A Ten‐Year‐Old Boy in Nanchong Shoulders the Household Alone, Studying While Caring for His Paralysed Father’ (People’s Dail and y Online 2014) portray children's caregiving as acts of heroic devotion rather than responses to structural neglect. These narratives depict child carers as *dongshi*, self‐sacrificing and *xiao* (filial), transforming emotional endurance into a celebrated public virtue. By glorifying perseverance and moral fortitude, such discourses obscure the material and emotional costs of care, reproducing the belief that family hardship should be resolved through individual morality and affective strength rather than through collective or institutional support. In this sense, public discourse functions as an affective amplifier: it turns children's private struggles into stories of moral triumph while depoliticising the structural conditions that necessitate their caregiving in the first place. While such narratives may foster pride and strengthen familial bonds, they also tether children to an emotional regime in which refusal risks moral condemnation, provoking shame or guilt.

### 6.2 Emotional Labour Theory: From Private Burden to Political Practice

This study extends Hochschild's (2012) concept of emotional labour beyond its established applications in paid employment and adult caregiving, aligning with a growing body of scholarship on non‐market forms of emotional labour within families and communities (e.g., English and Brown 2023; Ruiz 2024). While this literature has illuminated how affective work sustains interpersonal and kin relations, the emotional labour of children, particularly in contexts of welfare absence, remains underexplored. In the setting examined here, the ‘organisation’ is not a workplace but the family, and the implicit contract is moral rather than economic. Emotional labour is thus privatised, performed to sustain kin relations in the absence of adequate state provision. Yong's careful modulation of tone when speaking to his mother, and Liang's decision to withhold complaints while repairing household items, illustrate how such affective practices unfold as micro‐level processes of social reproduction. These are the everyday ways in which family life and wider social order are reproduced through care and emotional work, linking emotion management to the macro‐level lack of public welfare.

This reframing also reveals the double‐edged nature of emotional labour as both agency and constraint. Young carers often exercised tactical skill, delaying requests until a care recipient's mood improved or using humour to diffuse family tension. Yet this agency was bounded by gendered and generational hierarchies, as well as by the cultural imperative of filial piety. Xingxing's decision to ignore her foster mother's nocturnal outbursts, despite feeling guilty afterwards, exemplifies what Rai (2024) calls ‘constrained life‐making’: acting within, rather than beyond, the limits set by structural inequality. The same skills that sustained household relationships also deepened children's entanglement in caregiving arrangements they could not refuse, illustrating the paradoxical interplay between empowerment and entrapment.

### 6.3 Comparative Insights and Theoretical Implications

Situating these findings within the global literature on young carers reveals both shared patterns and context‐specific dynamics. Across settings from the UK to sub‐Saharan Africa, children's caregiving frequently arises in the shadow of inadequate public provision, with responsibilities disproportionately shouldered by children (Skovdal et al. 2009). Yet the emotional labour of Chinese young carers unfolds within a distinct moral and affective order. In Global North contexts such as the United States, Europe and Australia (Bjorgvinsdottir and Halldorsdottir 2014; Kavanaugh et al. 2016; Smyth et al. 2011), emotional labour is often framed as a psychological burden or welfare concern, emphasising the need for institutional recognition and support. By contrast, in China, moral idioms such as *xiao*, *chiku*, *dongshi* and *weiqu* recast similar affective efforts as signs of virtue and maturity rather than strain. These cultural norms fuse emotional restraint, endurance and gratitude into a normative ideal that narrows the space for articulating care in terms of fairness, redistribution or rights, making refusal or critique socially risky.

This difference highlights that, while the division of caregiving labour between adults and children is not unique to China, the moral grammar through which such labour is organised differs profoundly. The Chinese case demonstrates how emotional labour can be simultaneously universal and culturally particular—a shared human response to care demands, yet structured by locally specific feeling rules and welfare arrangements. In contexts where state support is limited and cultural norms legitimise endurance, children's emotional labour not only sustains family life but also substitutes for institutional care, turning private affect into an invisible form of social reproduction.

Framing emotional labour within the political economy of care extends ongoing sociological debates that treat care as both a moral practice and a site of inequality (Pettersen 2012; Tronto 2020). In post‐reform China, the retreat of state welfare has transformed families into ‘invisible welfare providers’. Within this arrangement, children's emotional labour operates as an unacknowledged subsidy to the welfare system. Liang's assumption of ‘man of the house’ duties, such as repairing appliances and maintaining household stability, exemplifies how such work absorbs the costs of care without recognition or compensation. This challenges sentimental portrayals of care as purely voluntary, instead revealing its entanglement with structural inequalities and policy neglect.

These insights also prompt a reconsideration of the normative boundaries of childhood. Dominant imaginaries depict childhood as a protected stage, buffered by adult care (Becker 2000; Rose and Cohen 2010). Yet the lived realities of young carers in China, and in many parts of the Global South such as Kenya (e.g., Skovdal et al. 2009), reveal childhood as a space where love, obligation and endurance coexist with sustained exposure to structural harm. Mengyuan's description of herself within the frame of the ‘good girl’ illustrates how cultural ideals can simultaneously affirm children's worth and constrain their emotional autonomy. Recognising these tensions contributes to a more inclusive sociology of childhood—one attentive to the affective norms that mediate children's caregiving experiences across diverse cultural and institutional contexts.

## 7 Conclusion

This study advances scholarship in two interconnected ways. First, it contributes to research on young carers by providing one of the few in‐depth accounts from China, where children's caregiving has been largely overlooked in both policy and academic discourse. By foregrounding how cultural norms such as filial piety, *dongshi*, and *chiku* shape children's affective practices, the study situates Chinese young carers within the wider international literature, challenging Global North‐centric understandings of childhood, caregiving, and vulnerability. Second, it extends emotional labour theory into non‐commodified, child‐performed care, integrating affective experiences into the analysis and demonstrating how emotional labour operates as a political practice that sustains, and at times contests, structural inequality.

Future research could undertake cross‐cultural comparisons to examine how affective norms shape young carers' experiences across different contexts. By foregrounding the everyday management of emotions such as weiqu and the striving for jinli, this study shows how children's emotional labour is organised within households as a private and intricate form of work. In doing so, it makes visible the privatised and complex nature of child carers' emotional labour, which is often obscured by idealised public and cultural discourses surrounding childhood and care in China. Recognising these dynamics reframes care as both a moral and a political practice and emphasises the need for policy interventions that acknowledge and support children, rather than relying on their invisible labour as a buffer for welfare retrenchment.

## Funding

This study is funded by British Association for International and Comparative Education (BAICE).

## Ethics Statements

This study has received ethics approval from a subcommittee of the University of Oxford Central University Research Ethics Committee (Ethics reference: R82187/RE001). All participants provided written informed consent prior to participating. Consent was also obtained from children's gatekeepers.

## Conflicts of Interest

The authors declared no potential conflicts of interest with respect to the research, authorship, and/or publication of this article.

## Permission to Reproduce Material From Other Sources

There is no reproduced material from other sources to declare.

## Data Availability

The data that support the findings of this study are available on request from the corresponding author. The data are not publicly available due to privacy or ethical restrictions.

## Endnotes

^1^

*Chiku* (吃苦), literally ‘to eat bitterness,’ is a culturally upheld ideal of endurance in Chinese moral life. As Arthur Kleinman explains, it refers not only to bearing hardship but to “keep going in spite of the fact that times are very hard,” embodying moral strength and acceptance of life's limits (Thomas et al. 2024, 36). In this study, the notion transforms suffering into moral virtue, legitimising perseverance as a sign of character and filial devotion.

^2^
Left‐behind children are those whose parents migrate for work, leaving them to live with grandparents or other relatives (Ye and Pan 2011).

^3^
Young carers in this study were taking on household management responsibilities to varying degrees. Tasks ranged from household repairs, shopping, heavy lifting, negotiating services for their parents or guardians, and actively searching for and providing helpful information and policy updates.

^4^
Several young carers provided assistance with routine daily activities, including helping care recipients to dress and undress, assisting with eating, administering or reminding them to take medication, prompting them to use the toilet, washing hair, wiping non‐intimate parts of the body, and related personal care tasks.

^5^

*Weiqu* (委屈) conveys a sense of grievance and moral restraint, encompassing feelings of being wronged yet obliged to remain silent in order to preserve relational harmony. It combines frustration, sadness and resignation, but also carries moral worth, as enduring unfairness without complaint is culturally idealised (Zou et al. 2020).

^6^

*Jinli* (尽力), literally ‘to exert one's utmost effort’, conveys a moral ideal of perseverance and responsibility rooted in traditional ethics of self‐cultivation and obligation (see Yan 2011). In children's caregiving, it reflects a culturally shaped sense of doing one's best despite fatigue or limited capacity, transforming emotional endurance into a measure of moral worth and filial devotion.

^7^
Left‐behind children are those whose parents migrate for work, leaving them to live with grandparents or other relatives (Ye and Pan 2011).

## Appendix I

**Table:**

Family case no.	Young carer (pseudonym)	Year of birth	Gender	Caregiving years	Type of school	Type of household	Family structure	Care recipient(s)	Diagnosis and/or disabilities	Primary caregiver	Other family member	Other co‐resident family members	Member who participated in the interview	Major income source	Main labour force’s occupation	External support
Participants’ Profile: C County, Family Cases No. 1 – 8																
1	Xiang	2007	Male	7	Regular senior secondary school	Marginal low‐income family	Lone‐parent family; single‐child family	Grandparents	Stroke and disabled (grandfather); physical pain (grandmother)	Xiang (the young carer)	Father	N/A	Grandmother	Grandmother	Farming	N/A
2	Jiaxin	2008	Male	7	Town‐level secondary school	Low‐income family	Lone‐parent family (after father died); multi‐child family	Mother	Congenital physical disability	Jiaxin (the young carer)	Older sister (born in 2002)	N/A	Mother	Mother	Doing odd jobs	N/A
3	Yifan	2007	Female	5	Vocational training high school	Low‐income family	Multi‐child family	Father	Thrombopenia; hypohepatia; renal insufficiency	Mother	Mother, older sister (born in 1997), grandmother	Mother, grandmother	Father	Mother	Working in a town‐level cake shop	Father’s younger brother (financially)
4	Qingyu	2007	Female	6	Vocational training high school	Low‐income family	Multi‐child family	Father	Chronic glomerulonephritis	Mother	Mother, older sister (born in 2000)	Mother	Mother	Mother	Working in a town‐level factory	N/A
5	Yong	2005	Male	11	Vocational training high school	Low‐income family	Multi‐child family	Parents	HIV/AIDS (both); cervical cancer and thyroid cancer (mother)	Yong (young carer)	Older sister (born in 1999)	N/A	Mother	Older sister	Working in a factory in Hangzhou city	N/A
6	Chao	2006	Male	4	Vocational training high school	Low‐income family	Lone‐parent; single‐child family	Mother	Breast cancer	Grandparents	Grandparents	Grandparents	Mother	N/A	N/A	N/A
7	Wenwen	2007	Female	7	Vocational training high school	Ordinary	Lone‐parent family; single‐child family	Father	Diabetes mellitus	Grandparents	Grandparents	Grandparents	Mother	Grandparents	Running a funeral shop	Father’s older brother (financially)
8	Lili	2007	Female	8	Vocational training high school	Low‐income family	Single‐child family	Parents	Peripheral neuropathy (father); myeloma (cancer), nephritic syndrome (mother)	Lili (young carer)	N/A	N/A	Father	N/A	N/A	Father’s older brother (help with farming)
Participants’ Profile: C County, Family Cases No. 9 – 15																
9	Longlong	2008	Male	5	Town‐level secondary school	Low‐income family	Multi‐child family	Parents	Acute myocardial infarction, high blood pressure, abnormal liver function (father); adenomyosis, ovarian tumor/cancer (mother)	Grandfather	Grandfather, older sister (born in 2003)	Grandfather	Grandfather	Mother	Working in a grocery store	Older sister (financially ‐ doing part‐time jobs)
10	Mengyuan	2005	Female	12	Vocational training high school	Marginal low‐income family	Lone‐parent family; single‐child family	Grandparents	Gastric cancer, high blood pressure, hyperlipidemia, diabetes mellitus (grandmother); dementia, physical disability (grandfather)	Mengyuan (young carer)	Father (congenital speech disability, doing odd jobs in Hangzhou city)	N/A	Grandmother	Mengyuan (young carer)	Doing odd jobs	Father’s younger sister (financially)
11	Jiajia	2010	Female	5	Town‐level secondary school	Low‐income family	Single‐child family	Grandmother	Cataract (blindness)/visual disability	Grandfather	N/A	Grandfather	Grandfather	Grandfather	Doing odd jobs	N/A
12	Chengcheng	2009	Female	6	Town‐level secondary school	Low‐income family	Multi‐child family	Mother, younger brother	Internal rheumatism (mother); nephrotic syndrome (younger brother)	Father	Grandmother	Father and grandmother	Father	Father	Village committee staff member	N/A
13	Zhenzhen	2008	Female	6	Town‐level secondary school	Low‐income family	Multi‐child family	Father	Uremia	Mother	Older sister (born in 1998)	Mother	Older Sister	Mother	Farming; working as a cleaner in the village	Older sister (financially ‐ working as a waitress in Hangzhou city)
14	Lele	2008	Male	9	Town‐level secondary school	Marginal low‐income family	Lone‐parent; multi‐child family	Older brother	Intellectual disability	Father	Grandfather	Grandfather	Father	Father	Doing odd jobs	Father’s older brother (caring for grandfather)
15	Qian	2006	Male	5	Vocational training high school	Ordinary	Lone‐parent family; single‐child family	Father	Stroke (paralysis)	Grandfather	Grandfather	Grandfather	Grandfather	Grandfather	Depending on pension	Nursing worker
Participants’ Profile: L District, Family Cases No. 1 – 8																
1	Xingxing	2006	Female	12	Vocational training high school	Low‐income family	Lone‐parent (after father died); adopted child, single‐child family	Mother	Epilepsy; mental disorder	Xingxing (young carer)	N/A	N/A	Mother	N/A	N/A	N/A
2	Dongdong	2008	Male	5	District‐level secondary school	Ordinary	Single‐child family	Father	Physical disability (lost right arm)	Mother	Mother	Mother	Mother	Mother	Insurance seller	N/A
3	Junjun	2010	Male	4	District‐level secondary school	Ordinary	Multi‐child family	Grandfather	Lung cancer	Grandmother	Parents, older brother (born in 2006)	Parents, grandmother	Mother	Parents	Courier (father); make‐up sales (mother)	N/A
4	Wei	2010	Male	3	District‐level secondary school	Ordinary	Multi‐child family	Mother	Breast cancer	Father	Older sister (born in 2009)	Father, older sister	Father	Father	Construction worker	N/A
5	Tianyu	2010	Male	7	District‐level secondary school	Marginal low‐income family	Single‐child family	Father	Diabetes mellitus	Mother	N/A	N/A	Mother	Mother	Glasses sales	N/A
6	Liang	2006	Male	4	Vocational training high school	Low‐income family	Lone‐parent (after father died); multi‐child family	Mother; grandmother	Cervical spondylosis (mother); physical disability, nephrotic syndrome (grandmother)	Liang (young carer)	Younger brother (born in 2020)	Younger brother	Mother	N/A	N/A	N/A
7	Leping	2007	Male	9	Vocational training high school	Low‐income family	Multi‐child family	Father; older sister	Liver cirrhosis, gastric cancer (father); congenital speech disability (older sister)	Mother	N/A	N/A	Father	Mother	Doing odd jobs	N/A
8	Yuqi	2011	Female	3	District‐level secondary school	Ordinary	Multi‐child family	Father	Renal failure	Grandmother	Mother, younger brother (born in 2014)	Mother, grandmother	Mother	Mother, grandmother	Massagist (mother); pension (grandmother)	N/A
Participants’ Profile: L District, Family Cases No. 9 – 15																
9	Xiyao	2010	Female	8	District‐level secondary school	Low‐income family	Lone‐parent (after father died); single‐child family	Mother	Congenital speech disability	Xiyao (young carer)	N/A	N/A	Grandmother	Mother	Doing odd jobs in factories	N/A
10	Yaqin	2009	Female	8	Vocational training high school	Low‐income family	Single‐child family; adopted child	Parents; grandparents	Congenital speech disability (parents); paralysis due to stroke (grandfather); breast cancer (grandmother)	Yaqin (primary carer)	N/A	N/A	Grandmother	Parents	Doing odd jobs in factories	N/A
11	Yao	2007	Female	6	Vocational training high school	Ordinary	Lone‐parent (divorced); adopted child, single‐chilld family	Grandmother	Diabetes mellitus	Yao (young carer)	Father (migrated for work)	N/A	Grandmother	Father	Construction worker	N/A
12	Chenxuan	2007	Female	2	Regular district‐level high school	Marginal low‐income family	Lone‐parent family; single‐child family	Grandfather	Uremia	Mother	N/A	N/A	Grandfather	Mother	Accountant	N/A
13	Feiyun	2008	Male	8	Vocational training high school	Low‐income family	Lone‐parent family; single‐child family	Father	Congenital vision disability	Feiyun (young carer)	N/A	N/A	Father	N/A	N/A	N/A
14	Maomao	2006	Female	5	Vocational training high school	Marginal low‐income family	Lone‐parent family;multi‐child family	Grandparents	Stroke (grandfather); breast cancer (grandmother)	Maomao (young carer)	Mother, younger brother (born in 2012)	Mother, younger brother	Mother	Mother	Insurance seller	N/A
15	Yueyue	2008	Male	9	Town‐level secondary school	Low‐income family	Lone‐parent family; single‐child family	Father; grandmother	Congenital speech disability (father); heart disease (grandmother)	Yueyue (young carer)	N/A	N/A	Grandmother	N/A	N/A	Father’s older sister (caring for grandmother)
